# Corrigendum to “A New Device Improves Signs and Symptoms of TMD”

**DOI:** 10.1155/2020/9465080

**Published:** 2020-04-17

**Authors:** Annalisa Monaco, Davide Pietropaoli, IAPNOR, Barry C. Cooper, Eleonora Ortu

**Affiliations:** ^1^University of L'Aquila, Department of Life, Health and Environmental Sciences, San Salvatore Hospital, Building Delta 6–Unit of Dentistry, V.le San Salvatore, L'Aquila 67100, Italy; ^2^I.A.P.N.O.R.—International Academy of Posture and Neuromyofascial Occlusion Research, Viale Gino Moretti 37, 63074 San Benedetto del Tronto (AP), Italy; ^3^Division of Translational Oral Biology, School of Dental Medicine, State University of New York, Stony Brook, New York, NY 14214, USA

In the article titled “A New Device Improves Signs and Symptoms of TMD” [1], we would like to clarify the title, the authorship, the provenance of the device with the International Academy of Posture and Neuromyofascial Occlusion Research (I.A.P.N.O.R.), and details of the methods. The article has been updated, and the original version is available in the supplementary materials.

The title has been revised to “A Device Improves Signs and Symptoms of TMD.” Ruggero Cattaneo and Dino Capparè have been removed from the author list, and I.A.P.N.O.R. has been added. Furthermore, the methods have been revised to accurately reflect the protocol for the clinical implementation of E.Li.Ba., aka ELIBA, as published by I.A.P.N.O.R. in 2010 [4].

The device is the Elevatore Linguale Balercia (E.Li.Ba, in English the Balercia Lingual Elevator), developed by the late Prof. Luigi Balercia, the founder of I.A.P.N.O.R., and described in 1998 [2] and 1999 [3]. The E.Li.Ba device was attributed to Prof. Balercia in the article, but the authors regret any implication that this was the first use or study of this device, that there was no reference to these publications or to I.A.P.N.O.R., and that the current membership of I.A.P.N.O.R. of the original last author Dr. Monaco and the previous membership of the original first author Dr. Cattaneo was not mentioned. Additional articles regarding E.Li.Ba were published in the proceedings of I.A.P.N.O.R.'s 2016–18 conferences by I.A.P.N.O.R.'s Futura Publishing Society.

We would also like to clarify that the study is part of the BENEFIT trial, which includes an additional arm on mandibular physiotherapy that is not yet published.

## References

[1] Monaco A., Pietropaoli D., IAPNOR, Cooper B. C., Ortu E. (2019). A new device improves signs and symptoms of TMD. *Pain Research and Management*.

[2] Balercia L., Serafini V. (1998). *Elevatore linguale Balercia, fisiopatologia della deglutizione*.

[3] Comparelli U. (1999). *Atlante ortodontico*.

[4] I.A.P.N.O.R (2010). *Protocollo IAPNOR per la realizzazione clinica dell’E.Li.Ba*.

