# Supplementary material for: Corrigendum to “A New Device Improves Signs and Symptoms of TMD”
Source: Pain Res Manag. 2020 Apr 17;2020:9465080. doi: 10.1155/2020/9465080 (PMC7201844; doi:10.1155/2020/9465080)
Supplement: Supplementary Materials — The previous version of the article is available as a PDF. [file 9465080.f1.pdf]

Hindawi
Pain Research and Management
Volume 2020, Article ID 9465080, 1 page
https://doi.org/10.1155/2020/9465080

Corrigendum

Corrigendum to "A New Device Improves Signs and Symptoms of TMD"

Annalisa Monaco,[1] **Davide Pietropaoli**,[1] **IAPNOR,**[2] **Barry C. Cooper,**[3] **and Eleonora Ortu**[1]

[1]*University of L'Aquila, Department of Life, Health and Environmental Sciences, San Salvatore Hospital, Building Delta 6–Unit of Dentistry, V.le San Salvatore, L'Aquila 67100, Italy*
[2]*I.A.P.N.O.R.—International Academy of Posture and Neuromyofascial Occlusion Research, Viale Gino Moretti 37, 63074 San Benedetto del Tronto (AP), Italy*
[3]*Division of Translational Oral Biology, School of Dental Medicine, State University of New York, Stony Brook, New York, NY 14214, USA*

Correspondence should be addressed to Eleonora Ortu; eleortu@gmail.com

Received 5 March 2020; Accepted 25 March 2020; Published 6 April 2020

Copyright © 2020 Annalisa Monaco et al. This is an open access article distributed under the Creative Commons Attribution License, which permits unrestricted use, distribution, and reproduction in any medium, provided the original work is properly cited.

In the article titled "A New Device Improves Signs and Symptoms of TMD" [1], we would like to clarify the title, the authorship, the provenance of the device with the International Academy of Posture and Neuromyofascial Occlusion Research (I.A.P.N.O.R.), and details of the methods. The article has been updated, and the original version is available in the supplementary materials.

The title has been revised to "A Device Improves Signs and Symptoms of TMD." Ruggero Cattaneo and Dino Capparè have been removed from the author list, and I.A.P.N.O.R. has been added. Furthermore, the methods have been revised to accurately reflect the protocol for the clinical implementation of E.Li.Ba., aka ELIBA, as published by I.A.P.N.O.R. in 2010 [4].

The device is the Elevatore Linguale Balercia (E.Li.Ba, in English the Balercia Lingual Elevator), developed by the late Prof. Luigi Balercia, the founder of I.A.P.N.O.R., and described in 1998 [2] and 1999 [3]. The E.Li.Ba device was attributed to Prof. Balercia in the article, but the authors regret any implication that this was the first use or study of this device, that there was no reference to these publications or to I.A.P.N.O.R., and that the current membership of I.A.P.N.O.R. of the original last author Dr. Monaco and the previous membership of the original first author Dr. Cattaneo was not mentioned. Additional articles regarding E.Li.Ba were published in the proceedings of I.A.P.N.O.R.'s 2016–18 conferences by I.A.P.N.O.R.'s Futura Publishing Society.

We would also like to clarify that the study is part of the BENEFIT trial, which includes an additional arm on mandibular physiotherapy that is not yet published.

Supplementary Materials

The previous version of the article is available as a PDF. (*Supplementary Materials*)

References

[1] A. Monaco, D. Pietropaoli, IAPNOR, B. C. Cooper, and E. Ortu, "A new device improves signs and symptoms of TMD," *Pain Research and Management*, vol. 2019, Article ID 5646143, 10 pages, 2019.

[2] L. Balercia, *Elevatore linguale Balercia, fisiopatologia della deglutizione*, V. Serafini, Ed., Futura Publishing Society, San Benedetto del Tronto, Italy, 1998, ISBN 9788887436037.

[3] U. Comparelli, *Atlante ortodontico*, Futura Publishing Society, San Benedetto del Tronto, Italy, 1999, ISBN 9788887436051.

[4] I.A.P.N.O.R , *Protocollo IAPNOR per la realizzazione clinica dell'E.Li.Ba*, Rivista IAPNOR, Supplement N. 1/2010, Futura Publishing Society, San Benedetto del Tronto, Italy, 2010.

Hindawi
Pain Research and Management
Volume 2019, Article ID 5646143, 10 pages
https://doi.org/10.1155/2019/5646143

Research Article

A New Device Improves Signs and Symptoms of TMD

Ruggero Cattaneo,[1] Davide Pietropaoli,[1] Barry C. Cooper,[2] Dino Capparè,[3] Eleonora Ortu,[1] and Annalisa Monaco[1]

[1]*University of L'Aquila, Department of Life, Health and Environmental Sciences, San Salvatore Hospital, Building Delta 6–Unit of Dentistry, V.le San Salvatore, L'Aquila 67100, Italy*
[2]*Division of Translational Oral Biology, School of Dental Medicine, State University of New York, Stony Brook, New York, NY 14214, USA*
[3]*Private Dental Technician, Dental Laboratory, Via Sallustio 4, 63074 San Benedetto Del Tronto (AP), Italy*

Correspondence should be addressed to Eleonora Ortu; eleortu@gmail.com

Received 19 November 2018; Revised 13 March 2019; Accepted 7 April 2019; Published 6 May 2019

Academic Editor: Marina De Tommaso

Copyright © 2019 Ruggero Cattaneo et al. This is an open access article distributed under the Creative Commons Attribution License, which permits unrestricted use, distribution, and reproduction in any medium, provided the original work is properly cited.

Background. Temporomandibular joint dysfunction (TMD) therapy remains an open challenge for modern dentistry. Herein, we propose a new neuromuscular lingual device able to reduce signs and symptoms of TMD in female patients with chronic orofacial pain. *Methods*. 50 females with myofascial TMD according to RDC/TMD were randomly assigned to study ($n = 25$) and control groups ($n = 25$). At T0, both groups received sEMG/KNG and pain evaluation by the VAS scale. The study group received the ELIBA device (lingual elevator by Balercia) constructed under ULF-TENS (ultra-low-frequency transcoutaneous electrical nervous stimulation). Subjects were instructed to use ELIBA at least for 16 h/day. After 6 months (T1), both groups underwent to sEMG/KNG and VAS revaluation. *Results*. T1 study group compared to controls showed a significant reduction in total ($p < 0.0001$) and mean ($p < 0.0001$) sEMG values, as well as a significant increase in both maximum vertical mouth opening ($p = 0.003$) and maximum velocity in mouth opening ($p = 0.003$) and closing ($p < 0.0001$). Interestingly, a significant reduction in pain measured by VAS ($p < 0.0001$) was reported. *Conclusions*. After 6 months, the ELIBA device is able to significantly reduce TMD-associated myogenous pain and to promote the enhancement of sEMG/KNG values. *Practical Implications*. ELIBA can be considered as a new device, potentially useful for head-neck pain relief in patients suffering from chronic TMD. In addition, its use promotes a muscles relaxation inducing freeway space increase. This characteristic makes it particularly useful for rehabilitation of patients with not enough space for construction of conventional orthotics or neuromuscular bites.

1. Introduction

Neuromuscular dentistry employs ultra-low-frequency transcutaneous electrical neural stimulation (ULF-TENS) to obtain a reduction of muscular tone utilizing surface electromyography (sEMG) to evaluate physiological freeway space [1–4]. Decrease of muscles' electrical resting hyperactivity and increase of interocclusal distance after ULF-TENS are necessary conditions to permit the fabrication of oral cavity devices, such as neuromuscular orthotics, which conform to the neuromuscular philosophy concepts and practices. Comfortable outcome for patients receiving this therapy has been documented [5]. In a small percentage of clinical cases (10–15%), it is not possible to get a reduction in electromyography resting electrical activity values and, above all, an increase of freeway space after ULF-TENS. This peculiar condition does not permit the use of neuromuscular orthotics to rehabilitate those patients [6]. One of the causes of failure to achieve reduction of electromyography values after ULF-TENS for some patients might be the lack of relaxation of suprahyoid muscles (digastric, stylohyoid, geniohyoid, and mylohyoid) and cervical muscles, which are agonists for mandibular and respiratory function [7]. In traditional orthodontic therapy, several devices have been proposed to improve tongue posture and function with the goal of modulating resting tongue position [8–11].

Corrected

Modification of tongue posture may entail an alteration of the neck and lower jaw posture in the rest position [12]. Among these devices, the lingual elevator (ELIBA) is the only one which employs ULF-TENS for its construction and permits the adaptation of the sublingual space (oral floor/suprahyoid muscles) specific to the anatomy and neuromuscular physiology of each individual. This procedure will be described in Methods [12–14]. The purpose of our research is to evaluate the effect of ELIBA (lingual elevator by Balercia) in patients suffering from temporomandibular disorders (TMDs) who did not experience either a significant reduction in electromyography resting values or an increase of interocclusal distance following ULF-TENS. A secondary goal of our work is to assess, with an individual scale, the positive effects on patient subjective symptoms after several months of therapy.

2. Materials and Methods

2.1. Subjects. This study was conducted in accordance with the Declaration of Helsinki. The Committee on Ethics in Science of the University of L'Aquila, L'Aquila, Italy, approved the study, and written informed consent was obtained from each subject and electronically stored as suggested by our institutional guidelines. This study was registered at: NCT02946645.

Fifty Caucasian patients (mean age 36.8; SD 8.5) who fulfilled the following criteria were included in the study group: (1) female gender; (2) age less than 50 years; (3) craniocervical myogenous TMD; (4) pain duration longer than 3 months; (5) reduction of freeway space and impairment sEMG activity after TENS according to Konchak et al. [6]; (6) presence of complete permanent dentition, with the possible exception of the third molars.

Patients were excluded from the study if they met one or more of the following criteria: (1) presence of systemic or metabolic diseases; (2) eye diseases or visual defects; (3) history of local or general trauma; (4) neurological or psychiatric disorders; (5) muscular diseases; (6) bruxism, as diagnosed by the presence of parafunctional facets and/or anamnesis of parafunctional tooth clenching and/or grinding; (7) pregnancy; (8) assumed use of anti-inflammatory, analgesic, antidepressant, opioid, or muscle relaxant medications; (9) smoking; (10) fixed or removable prostheses; (11) fixed restorations that affected the occlusal surfaces; and (12) either previous or concurrent orthodontic or orthognathic treatment [3].

The diagnosis of myofascial-type TMD was provided after clinical examination by a trained clinician according to group 1a and 1b of the Research Diagnostic Criteria for TMD (RDC/TMD), in a blinded manner (RC) [15, 16].

The enrolled subjects were randomly assigned to one of two groups: control group ($n = 25$) and study group ($n = 25$). The two groups were checked for age (study 36.41, SD 6.41; control 37.02, SD 9.15). Measurement of actual pain was recorded for each subject in a visual analog scale (VAS) of pain [17]. Each subject underwent sEMG and jaw tracking (KNG or computerize mandibular scan) recordings (TIME 0) according to the Monaco protocol in a blind fashion [7]. At the end of recordings, the study group underwent sublingual myoprint (sapphire® H Bosworth, Scokie, IL, USA) registration for the construction of the ELIBA device under TENS stimulation, while the control group did not. The study group received the device, and they were educated to use it at least sixteen hours a day. Checkups were carried out every 15 days for a total of six months. After six months, both control and study groups received a second sEMG/KNG (TIME 1) in a blind manner.

2.2. sEMG/Jaw Tracking Recording Procedure. Briefly, all examinations were performed using an 8-channel surface electromyograph with simultaneous acquisition, common grounding to all channels, and filters of 50 Hz. Data obtained were displayed and stored on an electromyography device (K7/EMG®, Myotronics-Noromed, Inc., Tukwila WA, USA), with disposable electrodes (Duotrode®, bipolar surface electrodes Ag-AgCl, 20 mm center to center distance, Myotronics-Noromed, Inc., Tukwila WA, USA), for sEMG recording. Resting electrical activity in the right masseter (RMM), left masseter (LMM), right anterior temporalis (RTA), left anterior temporalis (LTA), right digastric (RDA), left digastric (LDA), right sternocleidomastoid (RSCM), and left sternocleidomastoid (LSCM) muscles were recorded. The sEMG recordings and muscle activity was expressed as the root mean square (RMS) of the amplitude, expressed in μv [18]. Jaw tracking (Kinesiographic KNG) recordings were performed using a kinesiograph (K7/CMS®; Myotronics-Noromed, Inc., Tukwila, WA, USA) that measures jaw movements in three dimensions with an accuracy of 0.1 mm. A lightweight array weighing 113 grams containing 8 magnetic sensors affixed to the bridge of the nose and held in place with Velcro straps at the back of the skull tracked the motion of a 0.1oz magnet (CMS Magnet; Myotronics-Noromed, Inc., Tukwila WA, USA) that was attached to the labial gingiva beneath the mandibular incisor teeth in the mandibular midpoint with an adhesive gel. The kinesiography and electromyography were interfaced with a computer for data storage and subsequent software analysis (K7 Program, Myotronics-Noromed, Inc., Tukwila WA, USA).

Electrodes were positioned on LMM, RMM, LTA, and RTA, as previously described [19], as well on RDA, LDA [20], and LSC and RSC [21, 22]. A template was used to permit the exact repositioning of the electrodes on repeated testing sessions. The ground electrode, which was larger than the others and ensured a very good contact with the skin, was positioned on the subject's forehead to ensure a common reference to the differential input of the amplifier. The kinesiographic array was mounted on the bridge of the nose and aligned for the optimal position of the magnet for the recording of kinematic movements which was monitored by software. Inherent electromyographic noise was tested through K7 software for each channel and a value of 0.0 μv could be accepted. In case of excess of noise, a new electrode was placed in an appropriate location for that muscle.

sEMG/KNG recordings include the following:

(i) Scan 9—sEMG activity at basal condition with eyes lightly closed

Corrected

(ii) Scan 1—maximal voluntary mandibular opening
(iii) Scan 2—velocity of jaw movements during mandibular opening and closing
(iv) Scan 3—freeway space recording at the incisor point
(v) Motor trigeminal TENS stimulation
(vi) Scan 10—sEMG activity at basal condition with closed eyes after TENS
(vii) Scan 4—freeway space recording after TENS

Note: sEMG or KNG scans with artefacts due to swallowing or aberrant head or mandibular movements were discarded and the recordings were performed again.

2.3. TENS Stimulation Procedure. The method for sensory TENS was described previously [23–27]. Briefly, a J5 Myomonitor® TENS Unit device (Myotronics-Noromed, Inc., Tukwila, WA, USA) with disposable electrodes (Myotrode SG Electrodes®, Myotronics-Noromed, Inc., Tukwila, WA, USA) was used. This device generates a repetitive synchronous and bilateral stimulus delivered at 1.5-second intervals, with adjustable amplitude of approximately 0–24 mA, a duration of 500 μs, and a frequency of 0.66 Hz. The two TENS electrodes were placed bilaterally anterior to the tragus of each ear to provide neural stimulation of the mandibular division of the trigeminal nerve (CM V div 2). The electrode placement position was located between the coronoid and condylar processes of the mandible and was identified by manual palpation of the zone anterior to the tragus; a third common electrode was placed in the center of the back of the neck [3]. The amplitude of TENS stimulation started at 0 mA, with the stimulator device turned on and the rheostat, which controls the amplitude, positioned at 0. The amplitude of stimulation was progressively increased at a rate of 0.6 mA/s until the patients reported the sensation of pricking and the operator visualized and palpated the chin to confirm that a threshold stimulus causing a tiny rise of the chin was achieved. The operator delivered the TENS stimulation according to the manufacturer's guidelines. Test subjects were instructed to relax and permit their jaw to rise and fall without making tooth contacts.

2.4. ELIBA Construction. ELIBA is the acronym for elevator lingual by Balercia. Professor Luigi Balercia was the first to fabricate this device for orthodontic purposes (personal communication) in order to support the maintenance of the tongue posture in patients suffering from atypical swallowing after logopedic therapy. The appliance is designed to create an oral device that can be stably anchored in the lower arch (Figure 1). It involves the triangular shape of sublingual space delimited anteriorly and laterally by the mandible and lingual teeth surfaces, inferiorly by the oral floor (mainly constituted by mylohyoid muscle) and superiorly by the ventral surface of the tongue (Figure 1). For the purpose of taking an impression of sublingual space, prior stimulation with ULF-TENS is employed. The patient is asked to lean the apex of the tongue against the physiological spot (retroincisal papilla) and to maintain the tongue relaxed in this position for all the duration of the procedure. The subject is verbally instructed to protrude the mandible until the incisal edges of the upper and lower teeth are in contact, the patient being asked to maintain this position. After five minutes of sensorial ULF-TENS, the patient is asked to widely open mouth and impression material (Sapphire Resin, Myoprint) of plastic consistency is introduced with a proper syringe, in order to completely fill up sublingual space. The employed resin is the same impression material, with equal consistency, used to obtain myocentric (neuromuscular) bite registration in neuromuscular dentistry [28]. The subject is asked to close the mouth in the previously described position (upper and lower incisal edges in contact) and instructed to maintain the tongue relaxed with the apex against the physiological spot (retroincisal papilla). ULF-TENS amplitude is increased for some impulses to slightly above threshold level, until it is possible to observe lower jaw movements rising towards the upper arch. After some impulses (from 5 to 10), ULF-TENS amplitude is brought back down to the original threshold level, and it is necessary to wait for initial curing of impression resin to a resilient rubbery state. When resin has reached a firm but elastic consistency, before its complete hardening, it is taken out from oral cavity and put on the master model to finish its polymerization (Figure 1) [12, 13, 29]. The dental laboratory technique phases consist in duplicating the impression with proper material, inserting retentive anchors (Crozat clasps) on first mandibular molars and modelling a chrome cobalt thread of 1.2 millimeters of diameter on the lingual surfaces of the teeth in the lower arch to link together the resin anterior part of ELIBA and retentive anchors (Crozat clasps) which are on molars (Figure 1). ELIBA must not have occlusal contacts and should passively fit on the lower arch (Figure 1) [12].

3. Statistical Analysis

The statistical analysis was conducted using STATA 10® (StataCorp LP, College Station, TX, USA). Normal distribution of data was tested by the Shapiro–Wilk test. In order to compare within group the EMG-KIN and VAS data, the t-test for paired data was performed. The comparison between groups was carried out with t-test for unpaired data.

For EMG-mean parameter was calculated the algebraic mean of the sum of the mean rms of each muscle recorder according to Cooper [5]. The mean rms was automatically calculated by K7I program at the end of 15-second period of EMG recording.

For maximum opening (MO) of the mouth, maximum velocity of opening (MVO), and maximum velocity of closing (MVC), the mean of three consecutive movement cycles for each parameter was chosen.

Our hypothesis was that base data (TIME 0) did not differ significantly between the control and ELIBA groups, whereas the comparison of the two groups after therapeutic intervention at TIME 1 could differ if ELIBA, if the beneficial

Corrected

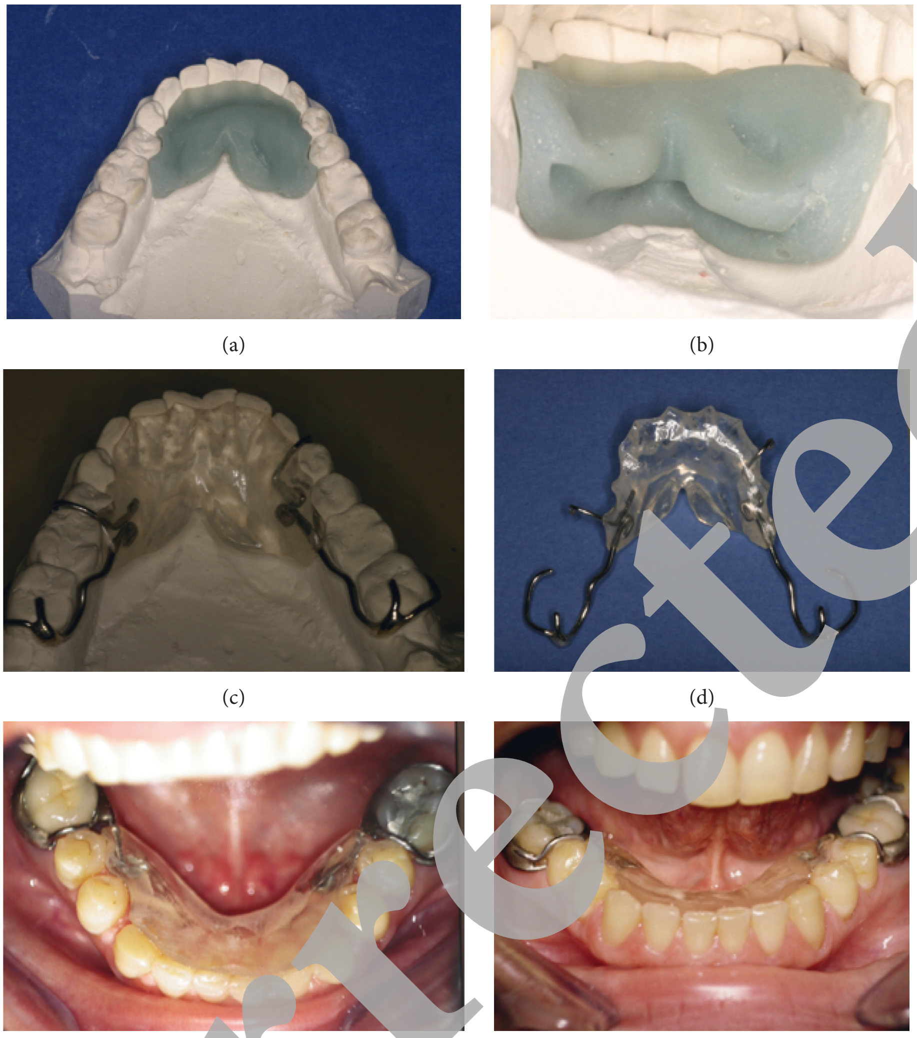

(a) (b) (c) (d) (e) (f)

Figure 1: ELIBA construction phases and oral aspect of the device. (a) Acrylic recording of the sublingual space; (b) detail of the caudal part of the acrylic impression; (c) the ELIBA appliance on the plastic cast; (d) ELIBA appliance; (e-f) intraoral views of the ELIBA appliance fitted to the oral cavity of the patient.

Corrected

effects of the appliance use were documented with EMG, KNG and VAS data obtained.

The level of significance was set at $p < 0.05$ for all tests. The results are expressed in terms of mean and standard deviation (SD), while in the bar plots, mean and standard error (SE) were represented.

4. Results

Tables 1 and 2 report the EMG/KNG comparability of the two groups in the base condition (TIME 0) according to EMG-KNG inclusion criteria.

Table 1 shows the EMG data of ELIBA and control groups in the base condition (TIME 0). No statistical significant reduction of values has been seen comparing before and after ULF-TENS in both groups (within-group comparison); for an immediate comparison, at a glance, see the value of EMG-mean: neither ELIBA (2.42 vs 2.16) nor control (2.13 vs 2.39) showed significant difference. No significance has been seen in EMG statistics comparing (between-group statistics) the two groups in the base condition (TIME 0).

The KNG measures of mandible velocity of opening and closure and the maximum opening of the mandible are plotted in Table 3. In TIME 0, no differences have been seen in the two groups. No difference between groups in VAS was noted (Tables 3 and 4). The EMG-KNG and VAS data in TIME 0 allowed the comparability of the two groups.

Table 2 refers to the KNG data and comparison of the freeway space (FWS) after TENS in TIME 0 and TIME 1. According to inclusion criteria, the mean values of vertical dimension (Vert.) of the FWS after ULF-TENS were lower than 1.5 mm in both groups. No significant differences were found between the two groups. In TIME 1, the FWS Vert. after ULF-TENS of the ELIBA group increases growing beyond the inclusion value of 1.5 mm. (0.92 vs 1.55;

Table 1: EMG values of ELIBA and control groups in the base condition (TIME 0) before and after ULF-TENS (S9 before ULF-TENS; S10 after ULF-TENS).

	ELIBA TIME 0			Control TIME 0			Statistics between the groups	
							Before TENS	After TENS
S9 (before TENS)	S10 (after TENS)	p	S9 (before TENS)	S10 (after TENS)	p	p	p	
LTA	2.98 (1.55)	1.73 (0.72)	**0.014**	2.17 (1.18)	2.00 (0.67)	0.576	0.121	0.290
LMM	1.68 (0.78)	1.05 (0.35)	**0.005**	1.69 (0.91)	1.23 (0.36)	0.117	0.966	0.161
RMM	1.65 (0.68)	1.67 (0.92)	0.930	1.38 (0.65)	1.71 (0.93)	0.335	0.283	0.907
RTA	2.55 (1.66)	2.19 (1.67)	0.393	2.05 (0.93)	2.96 (1.72)	0.055	0.313	0.226
LSC	3.08 (2.88)	3.38 (3.40)	0.768	2.93 (2.26)	3.19 (1.30)	0.688	0.878	0.839
LDA	2.07 (0.79)	2.43 (1.40)	0.212	1.87 (0.71)	2.34 (0.79)	0.125	0.487	0.825
RDA	2.19 (0.63)	2.05 (0.66)	0.519	2.10 (1.06)	2.33 (0.65)	0.427	0.787	0.253
RSC	3.18 (2.34)	2.82 (1.74)	0.522	2.83 (1.45)	3.38 (1.43)	0.307	0.630	0.344
sEMG-mean	2.42 (0.71)	2.16 (0.75)	0.272	2.13 (0.62)	2.39 (0.26)	0.104	0.238	0.283

Statistics: t-test for paired data for within-group comparison and for unpaired data in between-group comparison. For abbreviations, refer to Materials and Methods.

Table 2: Kinesiographic measurements and comparison of FWS (freeway space) after ULF-TENS in ELIBA and control groups in TIME 0 and TIME 1.

		ELIBA group			Control group			Statistics between the groups	
								TIME 0	TIME 1
	TIME 0	TIME 1	p	TIME 0	TIME 0	TIME 1	p	p	p
	Vert..	0.92 (0.47)	1.55 (0.73)	**0,011**	0.88 (0.28)	0.78 (0.15)	0,246	0.78	**0.001**
FWS after TENS	AP	0.65 (0.32)	0.99 (0.66)	0,092	0.76 (0.27)	0.75 (0.30)	0,900	0.31	0.220
	Lat.	0.41 (0.13)	0.39 (0.21)	0,919	0.41 (0.92)	0.54 (0.13)	**0,004**	0.74	**0.028**

Statistics: t-test for paired and unpaired data.

Table 3: KNG and VAS data and comparison between and within groups at TIME 0 and TIME 1.

	ELIBA group			Control group			Statistics between the groups	
							TIME 0	TIME 1
	TIME 0	TIME 1	p	TIME 0	TIME 1	p	p	p
MO	293.13 (44.26)	344.53 (35.95)	**0.002**	291.2 (35.26)	303.53 (31.51)	0.321	0.896	**0.003**
MVO	231.67 (64.32)	324.07 (98.51)	**0.006**	266.33 (55.83)	228.27 (55.54)	0.072	0.126	**0.003**
MVC	249.2 (57.48)	317.2 (37.44)	**0.001**	236.47 (44.14)	207.33 (35.07)	0.056	0.502	**≤0.001**
VAS	7.13 (1.06)	2.07 (1.22)	**≤0.001**	6.80 (0.94)	7.13 (0.92)	0.334	0.370	**≤0.001**

The EMG/KNG data used to support the findings of this study have not been made available because they are private data. Bold text represents statistical difference ($p < 0.05$). MO = maximum opening; MVO = maximum velocity of opening; MVC = maximum velocity of closing.

Table 4: sEMG values of ELIBA and control groups in TIME 1 before and after ULF-TENS.

	ELIBA TIME 1			Control TIME 1			Statistics between the groups	
							Before TENS	After TENS
	S9 (before TENS)	S10 (after TENS)	p	S9 (before TENS)	S10 (after TENS)	p	p	p
LTA	2.28 (0.99)	1.79 (0.85)	0.043	2.91 (0.61)	2.37 (0.71)	**0.009**	**0.049**	0.053
LMM	1.37 (0.39)	1.26 (0.57)	0.313	2.06 (0.74)	1.29 (0.27)	**0.003**	**0.004**	0.841
RMM	1.85 (1.09)	1.42 (0.66)	**0.008**	2.59 (0.68)	1.81 (0.64)	**0.005**	**0.034**	0.108
RTA	1.83 (0.49)	1.49 (0.65)	**0.004**	2.46 (0.94)	2.48 (1.52)	0.969	**0.031**	**0.032**
LSC	1.61 (0.48)	2.5 (1.75)	0.070	2.86 (1.25)	1.81 (0.91)	**0.033**	**0.002**	0.191
LDA	1.99 (0.66)	1.4 (0.57)	**<0.0001**	2.55 (0.79)	3.49 (1.32)	**0.023**	**0.042**	**≤0.001**
RDA	2.41 (0.33)	1.58 (0.72)	**<0.0001**	2.81 (0.56)	2.29 (0.59)	**0.027**	**0.025**	**0.006**
RSC	1.97 (0.89)	1.88 (1.17)	0.534	2.79 (1.22)	3.67 (1.42)	0.101	**0.045**	**0.001**
EMG-mean	1.91 (0.49)	1.67 (0.39)	**0.0001**	2.63 (0.44)	2.4 (0.41)	0.263	**≤0.001**	**≤0.001**

Statistics: t-test for paired data in within-group comparison and for unpaired data in between-group comparison. For abbreviations, refer to Materials and Methods. Bold text represents statistical difference ($p < 0.05$).

Corrected

$p = 0.011$). The control group does not increase the FWS vert. in TIME 1 comparing TIME 0. The between-group comparison indicates significant differences in vert. and lat. in TIME 1 (1.55 vs 0.78: $p = 0.001$ and 0.39 vs 0.54; $p = 0.028$, respectively).

Table 5 reports the EMG data recorded before ULF-TENS in TIME 0 and at the TIME 1 condition comparing the two groups. The data show that the ELIBA group demonstrates a reduction in the overall electrical activity in the rest condition (EMG-mean 2.42 vs 1.91; $p = 0.031$). This significance results by the sum of the reduction of the single muscles that individually are not able to reach the significance. The control group increases the overall rest EMG activity in TIME 1 (EMG-mean 2.13 vs 2.63; $p = 0.017$) because all muscles show higher resting electrical activity values in TIME 1 compared to TIME 0: LTA, RMM, LDA, and RDA increase reaches the significance in TIME 1 in the control group. The between-group comparison shows in TIME 0 no statistical significance in all muscles and EMG-mean, as already listed in Table 1. In TIME 1, all muscles and EMG-mean values are significantly lower in the ELIBA group compared to the control group.

Next, we compared the sEMG data before and after ULF-TENS within and between groups. As indicated in Table 5, the comparison between groups before ULF-TENS shows significantly lower resting electrical activity value in all muscles in the ELIBA patient group. After ULF-TENS, the data show that the most significant data are the decrease of EMG in the ELIBA group (1.67 vs 2.44; $p < 0.001$). The RTA, LDA, RDA, and RSC reached the level of significance between the two groups, lower in the ELIBA group than in the control.

Table 3 shows the KNG and VAS comparison between TIME 0 and TIME 1 between and within the groups. The ELIBA group increases in a significant manner all the KNG (maximum opening, maximum velocity of opening, and maximum velocity of closing) measures in TIME 1 compared to TIME 0. The VAS decreases significantly in this group in TIME 1 (7.13 vs 2.07; $p \leq 0.001$). The control group does not show differences in all KNG and VAS data comparing TIME 0 and TIME 1. The between-group comparison in TIME 1 indicates a significant difference in KNG and VAS measures, higher KNG and lower VAS values in ELIBA comparing control group.

5. Discussion

Data that were obtained from this study suggest that the use of ELIBA appliances in patients suffering from TMD

(1) significantly reduces resting electrical activity seen in sEMG values

(2) significantly increases both maximum vertical mouth opening and maximum velocity in mouth opening and closing values

(3) significantly increases the vertical component of the FWS after ULF-TENS

(4) significantly reduces pain

This oral device, initially born for orthodontic purposes, can help patients suffering from TMJ to relax the stomatognathic system (EMG and kinesiographic values). It can also help the patient in the reduction of the pain [12]. Usually electromyography values decrease and freeway space increases after ULF-TENS. When this phenomenon does not occur, in a few TMD patients, the clinician is faced with a big challenge in developing a diagnosis and a correct treatment planning in accordance with the neuromuscular gnathology philosophy and practices. However, assessing data of each research group, it was found that with this additional therapeutic aid, electromyography values tend to decrease after ULF-TENS [5,30–32].

Konchak in 1988 showed that in some patients, neither electromyography values decreased nor did interocclusal distance increase after ULF-TENS [6]. A few patients, 10%, did not relax neither chewing nor postural muscles after ULF-TENS. In 5% of subjects, freeway space even reduces. These patients cannot be treated pursuant to classic neuromuscular philosophy because ULF-TENS did not achieve muscle relaxation permitting the mandible to assume a physiologic mandible rest position. Moreover, lack of freeway space after ULF-TENS (less than one millimeter) does not permit the fabrication of neuromuscular orthotics that, even if very small, would further obliterate freeway space preventing the physiologic relaxation of muscles. Some authors have proposed the reduction of dental anatomy using burs in these TMD patients who do not respond favorably to ULF-TENS. However, unless patients already wear prosthetics, this solution is definitely discouraged, because it is not reversible and it does not assure a clinical beneficial outcome [33, 34]. The lower jaw rest position and consequent interocclusal freeway space can be partly influenced both by the tongue position between dental arches and by head and neck posture, mostly occurring through reciprocal interaction in essential functions such as breathing and swallowing.

The postural relationship between the hyoid, mandible, and neck is still controversial; Valenzuela et al. even state that there is no postural correlation [35]. Castro suggests a relation between sEMG activity of omohyoid muscle, which arises from the upper border of the scapula and inserts into the lower border of the body of the hyoid bone and anterior belly of digastric muscle during tongue movements and changes of position of the apex of the tongue [20]. Others found that the hyoid bone position generally had strong linear correlations with the positions of the head, jaw, and cervical vertebrae C1-C2 [36]. The cervical area between C1 and C3 receives proprioceptive afferent fibers from suboccipital, sternocleidomastoid, and trapezius muscles [37]. On the other hand, in healthy people, there is correlation between sEMG activity of sternocleidomastoid muscles and posture of the head and neck during physiological acts as swallowing and maximum voluntary clenching. This relationship tends to worsen in TMD patients [38].

In addition, as definitely demonstrated by Fitzgerald [39], the proprioception of the extrinsic and intrinsic muscles of the tongue, which are innervated with motor fibers by hypoglossal nerve (CN XII), is supplied by C1 and

Corrected

Table 5: Comparisons of TIME 0 vs TIME 1 of EMG values before ULF-TENS.

	ELIBA S9 (before TENS)			Control S9 (before TENS)			Statistics between the groups	
							TIME 0	TIME 1
	TIME 0	TIME 1	p	TIME 0	TIME 1	p	p	p
LTA	2.98 (1.55)	2.28 (0.99)	0.154	2.17 (1.18)	2.91 (0.61)	**0.045**	0.121	**0.049**
LMM	1.68 (0.78)	1.37 (0.39)	0.178	1.69 (0.91)	2.06 (0.74)	0.236	0.966	**0.004**
RMM	1.65 (0.68)	1.85 (1.09)	0.552	1.38 (0.65)	2.59 (0.68)	**≤0.001**	0.283	**0.034**
RTA	2.55 (1.66)	1.83 (0.49)	0.123	2.05 (0.93)	2.46 (0.94)	0.237	0.313	**0.031**
LSC	3.08 (2.88)	1.61 (0.48)	0.070	2.93 (2.26)	2.86 (1.25)	0.913	0.878	**0.002**
LDA	2.07 (0.79)	1.99 (0.66)	0.766	1.87 (0.71)	2.55 (0.79)	**0.020**	0.487	**0.042**
RDA	2.19 (0.63)	2.41 (0.33)	0.229	2.10 (1.06)	2.81 (0.56)	**0.031**	0.787	**0.025**
RSC	3.18 (2.34)	1.97 (0.89)	0.076	2.83 (1.45)	2.79 (1.22)	0.925	0.630	**0.045**
EMG-mean	2.42 (0.71)	1.91 (0.49)	**0.031**	2.13 (0.62)	2.63 (0.44)	**0.017**	0.238	**≤0.001**

Statistics: *t*-test for paired data in within-group comparison and for unpaired data in between-group comparison. For abbreviations, refer to Materials and Methods. Bold text represents statistical difference ($p < 0.05$).

C2 coming from the hypoglossal ansa sharing with the above cited muscles of neck. It seems probable that this correlation, that has anatomical and functional reasons, could have some clinical effect. For example, the position of hyoid, where extrinsic muscles of tongue are inserted, is correlated to tongue posture and to craniocervical angle: they both appear abnormal in sleep apnea patients in contrast to healthy subjects. Sleep apnea obstructive syndrome involves the tongue, lower jaw, pharynx, and neck in a pathophysiological way [40]. In sleep apnea patients, the mandibular position is abnormal, in part because during sleep, the tongue is more retruded than in healthy people [41, 42]. Functionally, the mandible, neck/head, and tongue are strictly associated in some reflex activities which induce a perpetual arrangement of reciprocal muscular tone [43, 44]. Stimulation of the lingual nerve, sensorial secondary branch of mandibular division of the trigeminal nerve (CN Vdiv3), determines the discharge of hypoglossal nerve fibers which concurrently cause tongue retrusion [45]. At the same time, opening the mouth determines the enhancement of sEMG activity of the genioglossus muscle and mandible and tongue posture can reciprocally influence [46]. For example, sensorial stimulation with light pressure stimulus of temporal muscle induces activation of motor neurons of hypoglossal nucleus, demonstrating a strict relationship between the postural muscles of the mandible and tongue [47]. The existence of an anatomical and functional relationship between muscles of neck and tongue has been experimentally documented by Edwards et al. [37]. In the intermediate nucleus of the medulla, the proprioception of suboccipital muscles, sternocleidomastoid, and trapezius is monosynaptical and related with the nucleus of the twelfth cranial nerves and with the nucleus of the solitary tract, this justifying the functional union among neck, head, and tongue and the vegetative answers inducted by postural variations of every element of this circuit. Among them are also some afferent fibers to the intermediate nucleus coming from vestibular and oculomotor nucleus [44]. It is also interesting to note that the tongue posture, position of apex of the tongue, and freeway space are correlated to watchfulness and mood. In fact, during watching emotional videos, the tension will be higher, the tongue posture will be lower, and interocclusal distance will be smaller [48]. The posture of apex of the tongue is related to other anatomical regions, specially the head and neck, and significantly influences orientation reactions in difficult visual search tasks [49].

The results of our work generally seem to be in accordance with concepts previously expressed. sEMG values at rest show a significant reduction of electrical potentials in muscles directly related with the lower jaw position (anterior temporalis and masseter muscle) and neck (sternocleidomastoid) making supposition that the ELIBA device action may partly be due both to sensorial stimulus to the lingual nerve and postural, determined by variation of position of tongue and apex of the tongue (C1-C2). This action may induce a different relation among all the parts related to the same system and involved in this phenomenon (head, neck, mandible, and tongue). Increase of kinesiography values of maximum mouth opening and maximum velocity in mouth opening can be interpreted as an enhancing in neuromuscular and sensorial balance [50].

In our work, we did not mathematically assess the position of tongue, apex of the tongue, and head and neck region, so we do not have data about spatial modifications of the anatomical regions involved in ELIBA. Our scientific assessment is limited to sEMG and kinesiography, so it is not excluded that more equilibrated muscles, and TMJ values can be obtained in the same spatial position of the head, neck, lower jaw, and tongue. However, even if data about it are not available, it is probable that an ELIBA appliance introduced in the sublingual space induces spatial variation of tongue position. About this assertion, the literature claims that introducing orthodontic devices in spaces useful for tongue determines modification of posture and functional movements of the tongue [30]. Employment of oral splints (orthoses) for TMD therapy still remains controversial because there is not a universal consensus as to diagnosis and pathogenesis of TMD [51–53]. Published studies assessing neuromuscular and not neuromuscular oral splints [5] have shown improvement of sEMG parameters and subjective symptoms. In our study, habitual occlusion has not been modified, and ELIBA use in fact does not require alteration

Corrected

Corrected

of preexistent occlusion. The purpose of our study was to demonstrate that it is possible to employ an oral device being able to modify tongue posture and ameliorate both symptoms and improve sEMG and KNG values in TMD patients whose sEMG and KNG parameters do not improve after ULF-TENS, and these patients cannot be included in a traditional neuromuscular gnathology protocol. It is relevant to specify that our results are not related with an occlusal modification such as occlusal adjustment according with neuromuscular or other gnathology philosophies. A limitation of our work is that it has not been effectuated a comparison with a control placebo group. We cannot assess how much of the positive clinical outcome may be due to placebo effect. A future paper will compare ELIBA clinical results with the outcome obtained with a neuromuscular occlusal splint and with placebo therapy [2, 3, 5, 54].

Data Availability

The EMG/KNG data used to support the findings of this study have not been made available because they are private data.

Conflicts of Interest

The authors declare that there are no conflicts of interest.

References

[1] B. Jankelson, “Three-dimensional orthodontic diagnosis and treatment. A neuromuscular approach,” *Journal of Clinical Orthodontics: JCO*, vol. 18, no. 9, pp. 627–36, 1984.

[2] B. C. Cooper, “More about TMD and SEMG,” *Journal of the American Dental Association*, vol. 137, no. 10, pp. 1362-1363, 2006.

[3] A. Monaco, R. Cattaneo, L. Mesin, E. Ortu, M. Giannoni, and D. Pietropaoli, “Dysregulation of the descending pain system in temporomandibular disorders revealed by low-frequency sensory transcutaneous electrical nerve stimulation: a pupillometric study,” *PloS One*, vol. 10, no. 4, Article ID e0122826, 2015.

[4] S. Tecco, S. Mummolo, E. Marchetti et al., “sEMG activity of masticatory, neck, and trunk muscles during the treatment of scoliosis with functional braces. A longitudinal controlled study,” *Journal of Electromyography and Kinesiology*, vol. 21, no. 6, pp. 885–892, 2011.

[5] B. C. Cooper and I. Kleinberg, “Establishment of a temporomandibular physiological state with neuromuscular orthosis treatment affects reduction of TMD symptoms in 313 patients,” *CRANIO®*, vol. 26, no. 2, pp. 104–117, 2008.

[6] P. A. Konchak, N. R. Thomas, D. T. Lanigan, and R. M. Devon, “Freeway space measurement using mandibular kinesiograph and EMG before and after TENS,” *Angle Orthodontist*, vol. 58, no. 4, pp. 343–50, 1988.

[7] A. Monaco and R. Cattaneo, *Elettromiografia e kinesiografia, Per la Clinica Odontoiatrica: Principi di Odontoiatria Neuro Mio Fasciale*, Libreria Cortina S.R.L., Milan, Italy, 2007.

[8] R. Condò, M. Costacurta, C. Perugia, and R. Docimo, “A typical deglutition: diagnosis and interceptive treatment: a clinical study,” *European Journal of Paediatric Dentistry: Official Journal of European Academy of Paediatric Dentistry*, vol. 13, no. 3, pp. 209–14, 2012.

[9] D. Ciavarella, L. Lo Russo, M. Mastrovincenzo et al., “Cephalometric evaluation of tongue position and airway remodelling in children treated with swallowing occlusal contact intercept appliance (S.O.C.I.A.),” *International Journal of Pediatric Otorhinolaryngology*, vol. 78, no. 11, pp. 1857–1860, 2014.

[10] E. Ortu, D. Pietropaoli, M. Ortu, M. Giannoni, and A. Monaco, “Evaluation of cervical posture following rapid maxillary expansion: a review of literature,” *The Open Dentistry Journal*, vol. 8, no. 1, pp. 20–27, 2014.

[11] E. Ortu, M. Giannoni, M. Ortu, R. Gatto, and A. Monaco, “Oropharyngeal airway changes after rapid maxillary expansion: the state of the art,” *International Journal of Clinical and Experimental Medicine*, vol. 7, no. 7, pp. 1632–8, 2014.

[12] E. Ortu, D. Pietropaolio, R. Cattaneo, M. Giannoni, and A. Monaco, “Overjet reduction with the use of the ELIBA device: a case report,” *Journal of Clinical and Diagnostic Research*, vol. 12, no. 12, pp. 8–10, 2018.

[13] F. L. N. Montagna, V. Piras, G. Denotti, and E. I. S. U. O. I. L’ortodonzia, *Dispositivi. Apparecchi Mobili E Fissi Rimovibili Nella Pratica Clinica. Orthodontics, fixed and removable devices in clinical practice*, 2007.

[14] S. Tecco, A. Baldini, S. Mummolo et al., “Frenulectomy of the tongue and the influence of rehabilitation exercises on the sEMG activity of masticatory muscles,” *Journal of Electromyography and Kinesiology*, vol. 25, no. 4, pp. 619–628, 2015.

[15] S. F. Dworkin, “Research diagnostic criteria for temporomandibular disorders: current status & future relevance1,” *Journal of Oral Rehabilitation*, vol. 37, no. 10, pp. 734–743, 2010.

[16] E. Schiffman, R. Ohrbach, E. Truelove et al., “Diagnostic criteria for temporomandibular disorders (DC/TMD) for clinical and research applications: recommendations of the International RDC/TMD Consortium Network and Orofacial Pain Special Interest Group,” *Journal of Oral & Facial Pain and Headache*, vol. 28, no. 1, pp. 6–27, 2014.

[17] H. M. Michelotti, D. J. de L. Horne, and S. Sheather, “Clinical applications of visual analogue scales: a critical review,” *Psychological Medicine*, vol. 18, no. 4, pp. 1007–1019, 1988.

[18] A. van der Bilt, F. G. Weijnen, F. Bosman, H. W. van der Glas, and J. B. M. Kuks, “Controlled study of EMG activity of the jaw closers and openers during mastication in patients with myasthenia gravis,” *European Journal of Oral Sciences*, vol. 109, no. 3, pp. 160–164, 2001.

[19] T. Castroflorio, D. Farina, A. Bottin, M. G. Piancino, P. Bracco, and R. Merletti, “Surface EMG of jaw elevator muscles: effect of electrode location and inter-electrode distance,” *Journal of Oral Rehabilitation*, vol. 32, no. 6, pp. 411–417, 2005.

[20] H. A. L. Castro, L. A. L. Resende, F. Bérzin, and B. König, “Electromyographic analysis of the superior belly of the omohyoid muscle and anterior belly of the digastric muscle in tongue and head movements,” *Journal of Electromyography and Kinesiology*, vol. 9, no. 3, pp. 229–232, 1999.

[21] D. Falla, P. Dall’Alba, A. Rainoldi, R. Merletti, and G. Jull, “Location of innervation zones of sternocleidomastoid and scalene muscles—a basis for clinical and research electromyography applications,” *Clinical Neurophysiology*, vol. 113, no. 1, pp. 57–63, 2002.

[22] D. Falla, P. Dall’Alba, A. Rainoldi, R. Merletti, and G. Jull, “Repeatability of surface EMG variables in the sternocleidomastoid and anterior scalene muscles,” *European Journal of Applied Physiology*, vol. 87, no. 6, pp. 542–549, 2002.

[23] A. Monaco, R. Cattaneo, L. Mesin, I. Ciarrocchi, F. Sgolastra, and D. Pietropaoli, "Dysregulation of the autonomous nervous system in patients with temporomandibular disorder: a pupillometric study," *PLoS One*, vol. 7, no. 9, Article ID e45424, 2012.

[24] E. Ortu, D. Pietropaoli, G. Mazzei, R. Cattaneo, M. Giannoni, and A. Monaco, "TENS effects on salivary stress markers: a pilot study," *International Journal of Immunopathology and Pharmacology*, vol. 28, no. 1, pp. 114–118, 2015.

[25] A. Monaco, R. Cattaneo, E. Ortu, M. V. Constantinescu, and D. Pietropaoli, "Sensory trigeminal ULF-TENS stimulation reduces HRV response to experimentally induced arithmetic stress: a randomized clinical trial," *Physiology & Behavior*, vol. 173, pp. 209–215, 2017.

[26] E. Ortu, D. Pietropaoli, F. Adib, C. Masci, M. Giannoni, and A. Monaco, "Electromyographic evaluation in children orthodontically treated for skeletal Class II malocclusion: Comparison of two treatment techniques," *Cranio-Journal of Craniomandibular Practice*, vol. 37, no. 2, pp. 129–135, 2019.

[27] S. Mummolo, A. Nota, S. Tecco et al., "Ultra-low-frequency transcutaneous electric nerve stimulation (ULF-TENS) in subjects with craniofacial pain: a retrospective study," *Cranio*, pp. 1–6, 2018.

[28] B. C. Cooper, "The role of bioelectronic instruments in documenting and managing temporomandibular disorders," *Journal of the American Dental Association*, vol. 127, no. 11, pp. 1611–1614, 1996.

[29] Y. S. Kim, S. Y. Kown, Y. G. Park, and K. R. Chung, "Clinical application of the tongue elevator," *Journal of Clinical Orthodontics: JCO*, vol. 36, no. 2, pp. 104–6, 2002.

[30] A. Monaco, F. Sgolastra, D. Pietropaoli, M. Giannoni, and R. Cattaneo, "Comparison between sensory and motor transcutaneous electrical nervous stimulation on electromyographic and kinesiographic activity of patients with temporomandibular disorder: a controlled clinical trial," *BMC Musculoskeletal Disorders*, vol. 14, no. 1, p. 168, 2013.

[31] A. Monaco, F. Sgolastra, I. Ciarrocchi, and R. Cattaneo, "Effects of transcutaneous electrical nervous stimulation on electromyographic and kinesiographic activity of patients with temporomandibular disorders: a placebo-controlled study," *Journal of Electromyography and Kinesiology*, vol. 22-, no. 3, pp. 463–468, 2012.

[32] N. Chipaila, F. Sgolastra, A. Spadaro et al., "The effects of ULF-TENS stimulation on gnathology: the state of the art," *Cranio*, vol. 32, no. 2, pp. 118–130, 2014.

[33] T. I. Suvinen and P. Kemppainen, "Review of clinical EMG studies related to muscle and occlusal factors in healthy and TMD subjects," *Journal of Oral Rehabilitation*, vol. 34, no. 9, pp. 631–644, 2007.

[34] A. Monaco, R. Cattaneo, M. C. Marci, D. Pietropaoli, and E. Ortu, "Central sensitization-based classification for temporomandibular disorders: a pathogenetic hypothesis," *Pain Research & Management*, vol. 2017, Article ID 5957076, 13 pages, 2017.

[35] S. Valenzuela, R. Miralles, M. J. Ravera et al., "Does head posture have a significant effect on the hyoid bone position and sternocleidomastoid electromyographic activity in young adults?," *Cranio*, vol. 23, no. 3, pp. 204–211, 2005.

[36] L. Zheng, J. Jahn, and A. N. Vasavada, "Sagittal plane kinematics of the adult hyoid bone," *Journal of Biomechanics*, vol. 45, no. 3, pp. 531–536, 2012.

[37] I. J. Edwards, V. K. Lall, J. F. Paton et al., "Neck muscle afferents influence oromotor and cardiorespiratory brainstem neural circuits," *Brain Structure and Function*, vol. 220, no. 3, pp. 1421–1436, 2015.

[38] H. Santander, R. Miralles, J. Pérez et al., "Effects of head and neck inclination on bilateral sternocleidomastoid EMG activity in healthy subjects and in patients with myogenic cranio-cervical-mandibular dysfunction," *Cranio*, vol. 18, no. 3, pp. 181–191, 2000.

[39] M. J. Fitzgerald and S. R. Sachithanandan, "The structure and source of lingual proprioceptors in the monkey," *Journal of Anatomy*, vol. 128, no. Pt 3, pp. 523–52, 1979.

[40] D. Passali, M. C. Spinosi, and F. M. Passali, "Ear nose and throat (ENT) aspects of obstructive sleep apnea syndrome (OSAS) diagnosis and therapy," *La Medicina del Lavoro*, vol. 108, no. 4, pp. 288–292, 2017.

[41] D. Passali, G. Corallo, A Petti et al., "A comparative study on oxidative stress role in nasal breathing impairment and obstructive sleep apnoea syndrome," *Acta Otorhinolaryngologica Italica: Organo Ufficiale Della Societa Italiana di Otorinolaringologia e Chirurgia Cervico-Facciale*, vol. 36, no. 6, pp. 490–495, 2016.

[42] K. Miyamoto, M. M. Özbek, A. A. Lowe et al., "Mandibular posture during sleep in patients with obstructive sleep apnoea," *Archives of Oral Biology*, vol. 44, no. 8, pp. 657–664, 1999.

[43] A. J. Miller, "Oral and pharyngeal reflexes in the mammalian nervous system: their diverse range in complexity and the pivotal role of the tongue," *Critical Reviews in Oral Biology and Medicine*, vol. 13, no. 5, pp. 409–425, 2002.

[44] N. Marchili, E. Ortu, D. Pietropaoli, R. Cattaneo, and A. Monaco, "Dental occlusion and ophthalmology: a literature review," *The Open Dentistry Journal*, vol. 10, no. 1, pp. 460–468, 2016.

[45] M. Takata, "Lingually induced inhibitory postsynaptic potentials in hypoglossal motoneurons after axotomy," *Brain Research*, vol. 224, no. 1, pp. 165–169, 1981.

[46] A. A. Lowe, S. C. Gurza, and B. J. Sessle, "Regulation of genioglossus and masseter muscle activity in man," *Archives of Oral Biology*, vol. 22, no. 10-11, pp. 579–584, 1977.

[47] Y. Ishiwata, T. Ono, T. Kuroda, and Y. Nakamura, "Jaw-tongue reflex: afferents, central pathways, and synaptic potentials in hypoglossal motoneurons in the cat," *Journal of Dental Research*, vol. 79, no. 8, pp. 1626–1634, 2000.

[48] P. Bourdiol, A. Mishellany-Dutour, M.-A. Peyron, and A. Woda, "Mood-induced variations of mandible and tongue postures," *Journal of Oral Rehabilitation*, vol. 40, no. 6, pp. 443–449, 2013.

[49] M. Barnett-Cowan, M. Soeizi, and J. F. X. DeSouza, "Visual attention at the tip of the tongue," *i-Perception*, vol. 6, no. 1, pp. 1–4, 2015.

[50] A. Monaco, V. Cozzolino, R. Cattaneo, T. Cutilli, and A. Spadaro, "Osteopathic manipulative treatment (OMT) effects on mandibular kinetics: kinesiographic study," *European Journal of Paediatric Dentistry: Official Journal of European Academy of Paediatric Dentistry*, vol. 9, no. 1, pp. 37–42, 2008.

[51] G. D. Klasser and C. S. Greene, "Oral appliances in the management of temporomandibular disorders," *Oral Surgery, Oral Medicine, Oral Pathology, Oral Radiology, and Endodontology*, vol. 107, no. 2, pp. 212–223, 2009.

[52] Y. Tsukiyama, K. Baba, and G. T. Clark, "An evidence-based assessment of occlusal adjustment as a treatment for temporomandibular disorders," *Journal of Prosthetic Dentistry*, vol. 86, no. 1, pp. 57–66, 2001.

Corrected

[53] S. S. Adibi, E. I. Ogbureke, B. B. Minavi, and K. U. Ogbureke, "Why use oral splints for temporomandibular disorders (TMDs)?," *Texas Dental Journal*, vol. 131, no. 6, pp. 450–455, 2014.

[54] B. C. Cooper, "TMD diagnostics," *Journal of the American Dental Association*, vol. 144, no. 9, pp. 980–982, 2013.

Corrected
